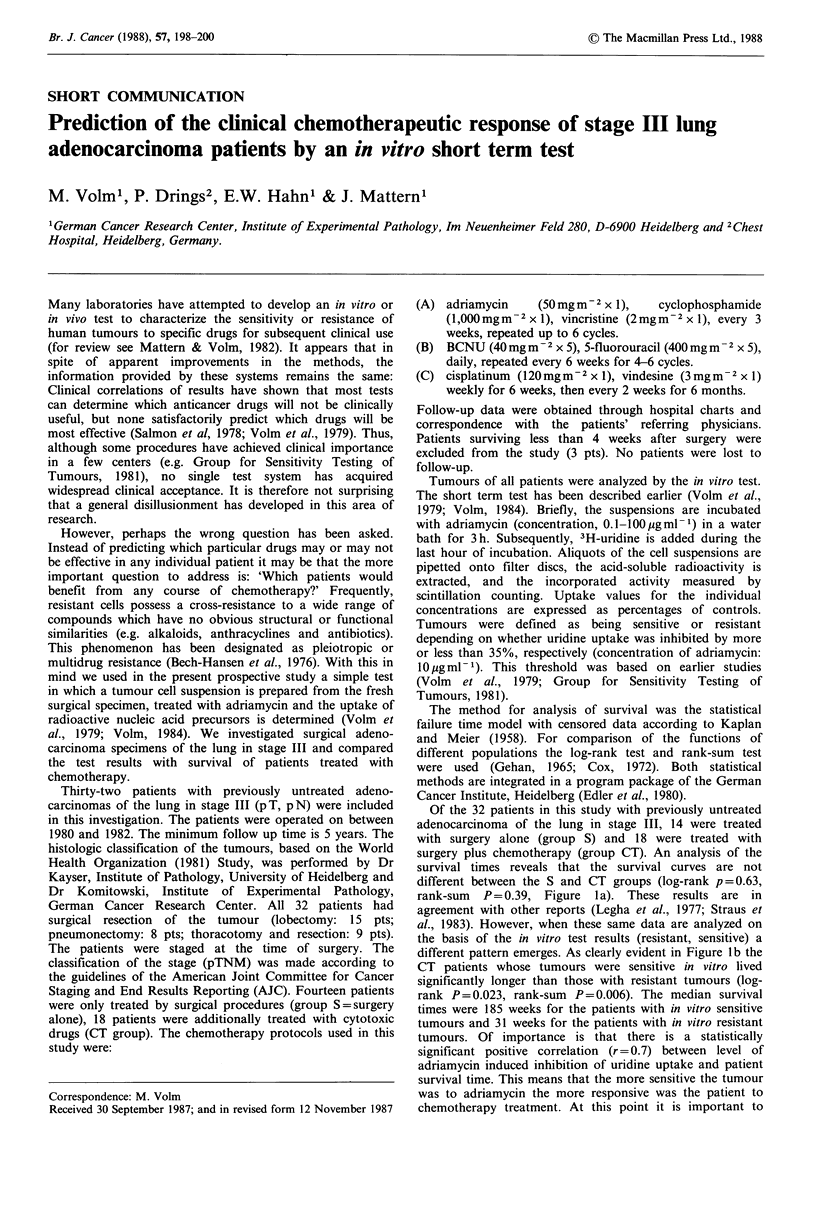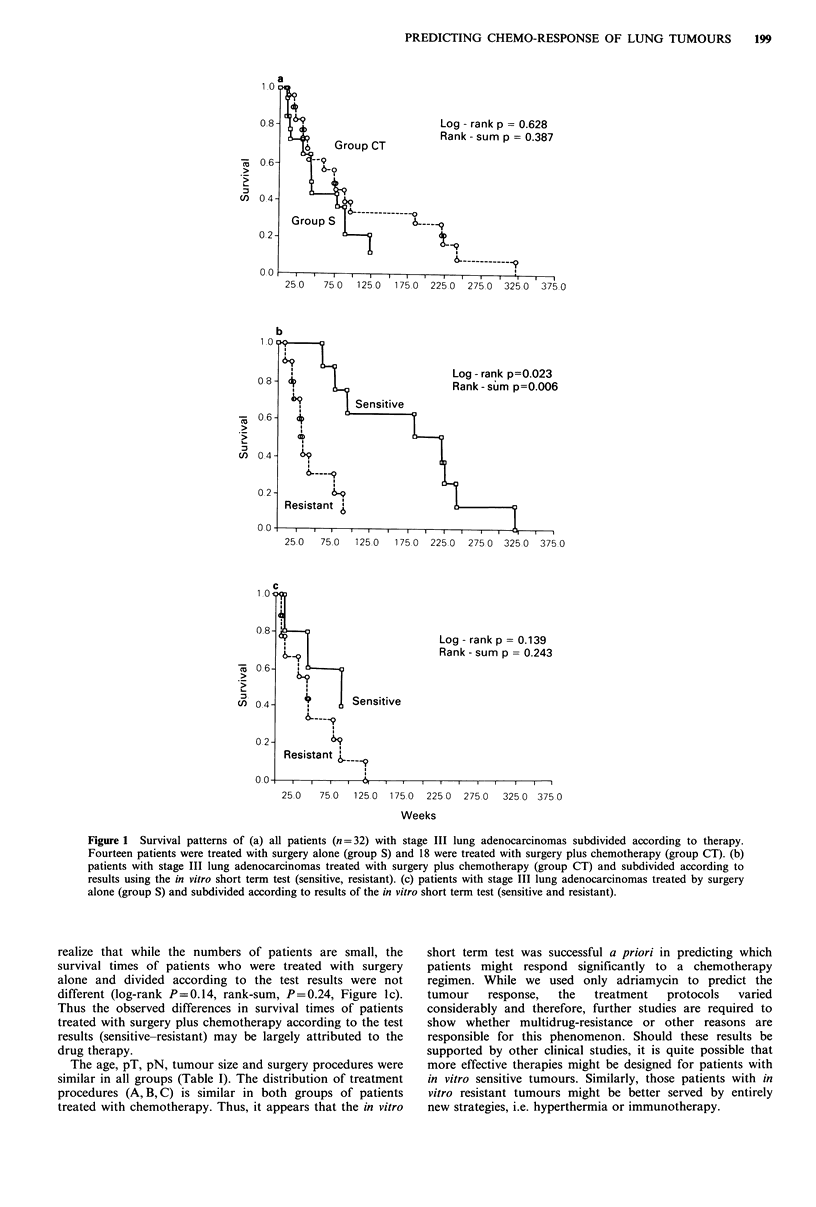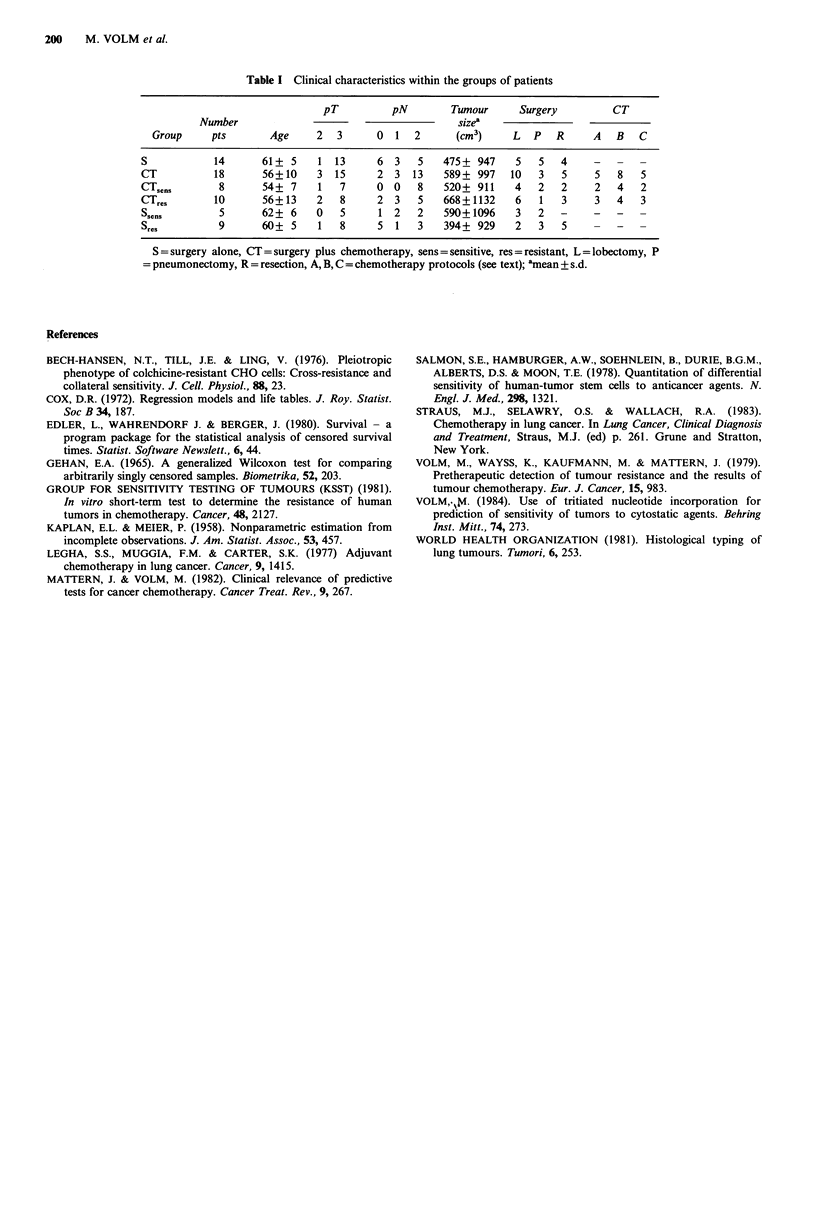# Prediction of the clinical chemotherapeutic response of stage III lung adenocarcinoma patients by an in vitro short term test.

**DOI:** 10.1038/bjc.1988.42

**Published:** 1988-02

**Authors:** M. Volm, P. Drings, E. W. Hahn, J. Mattern

**Affiliations:** German Cancer Research Center, Institute of Experimental Pathology, Heidelberg, Germany.


SHORT COMMUNICATION

### Prediction of the clinical chemotherapeutic response of stage III lung adenocarcinoma patients by an *in vitro* short term test

M. Volm[1], P. Drings[2], E.W. Hahn[1] & J. Mattern[1]

[1]*German Cancer Research Center, Institute of Experimental Pathology, Im Neuenheimer Feld 280, D-6900 Heidelberg and* [2]*Chest Hospital, Heidelberg, Germany.*

Many laboratories have attempted to develop an *in vitro* or *in vivo* test to characterize the sensitivity or resistance of human tumours to specific drugs for subsequent clinical use (for review see Mattern & Volm, 1982). It appears that in spite of apparent improvements in the methods, the information provided by these systems remains the same: Clinical correlations of results have shown that most tests can determine which anticancer drugs will not be clinically useful, but none satisfactorily predict which drugs will be most effective (Salmon *et al*, 1978; Volm *et al.*, 1979). Thus, although some procedures have achieved clinical importance in a few centers (e.g. Group for Sensitivity Testing of Tumours, 1981), no single test system has acquired widespread clinical acceptance. It is therefore not surprising that a general disillusionment has developed in this area of research.

However, perhaps the wrong question has been asked. Instead of predicting which particular drugs may or may not be effective in any individual patient it may be that the more important question to address is: 'Which patients would benefit from any course of chemotherapy?' Frequently, resistant cells possess a cross-resistance to a wide range of compounds which have no obvious structural or functional similarities (e.g. alkaloids, anthracyclines and antibiotics). This phenomenon has been designated as pleiotropic or multidrug resistance (Bech-Hansen *et al.*, 1976). With this in mind we used in the present prospective study a simple test in which a tumour cell suspension is prepared from the fresh surgical specimen, treated with adriamycin and the uptake of radioactive nucleic acid precursors is determined (Volm *et al.*, 1979; Volm, 1984). We investigated surgical adenocarcinoma specimens of the lung in stage III and compared the test results with survival of patients treated with chemotherapy.

Thirty-two patients with previously untreated adenocarcinomas of the lung in stage III (pT, pN) were included in this investigation. The patients were operated on between 1980 and 1982. The minimum follow up time is 5 years. The histologic classification of the tumours, based on the World Health Organization (1981) Study, was performed by Dr Kayser, Institute of Pathology, University of Heidelberg and Dr Komitowski, Institute of Experimental Pathology, German Cancer Research Center. All 32 patients had surgical resection of the tumour (lobectomy: 15 pts; pneumonectomy: 8 pts; thoracotomy and resection: 9 pts). The patients were staged at the time of surgery. The classification of the stage (pTNM) was made according to the guidelines of the American Joint Committee for Cancer Staging and End Results Reporting (AJC). Fourteen patients were only treated by surgical procedures (group S = surgery alone), 18 patients were additionally treated with cytotoxic drugs (CT group). The chemotherapy protocols used in this study were:

(A) adriamycin ($50\,mg\,m^{-2} \times 1$), cyclophosphamide ($1{,}000\,mg\,m^{-2} \times 1$), vincristine ($2\,mg\,m^{-2} \times 1$), every 3 weeks, repeated up to 6 cycles.

(B) BCNU ($40\,mg\,m^{-2} \times 5$), 5-fluorouracil ($400\,mg\,m^{-2} \times 5$), daily, repeated every 6 weeks for 4–6 cycles.

(C) cisplatinum ($120\,mg\,m^{-2} \times 1$), vindesine ($3\,mg\,m^{-2} \times 1$) weekly for 6 weeks, then every 2 weeks for 6 months.

Follow-up data were obtained through hospital charts and correspondence with the patients' referring physicians. Patients surviving less than 4 weeks after surgery were excluded from the study (3 pts). No patients were lost to follow-up.

Tumours of all patients were analyzed by the *in vitro* test. The short term test has been described earlier (Volm *et al.*, 1979; Volm, 1984). Briefly, the suspensions are incubated with adriamycin (concentration, $0.1$–$100\,\mu g\,ml^{-1}$) in a water bath for 3 h. Subsequently, $^{3}H$-uridine is added during the last hour of incubation. Aliquots of the cell suspensions are pipetted onto filter discs, the acid-soluble radioactivity is extracted, and the incorporated activity measured by scintillation counting. Uptake values for the individual concentrations are expressed as percentages of controls. Tumours were defined as being sensitive or resistant depending on whether uridine uptake was inhibited by more or less than 35%, respectively (concentration of adriamycin: $10\,\mu g\,ml^{-1}$). This threshold was based on earlier studies (Volm *et al.*, 1979; Group for Sensitivity Testing of Tumours, 1981).

The method for analysis of survival was the statistical failure time model with censored data according to Kaplan and Meier (1958). For comparison of the functions of different populations the log-rank test and rank-sum test were used (Gehan, 1965; Cox, 1972). Both statistical methods are integrated in a program package of the German Cancer Institute, Heidelberg (Edler *et al.*, 1980).

Of the 32 patients in this study with previously untreated adenocarcinoma of the lung in stage III, 14 were treated with surgery alone (group S) and 18 were treated with surgery plus chemotherapy (group CT). An analysis of the survival times reveals that the survival curves are not different between the S and CT groups (log-rank $p = 0.63$, rank-sum $P = 0.39$, Figure 1a). These results are in agreement with other reports (Legha *et al.*, 1977; Straus *et al.*, 1983). However, when these same data are analyzed on the basis of the *in vitro* test results (resistant, sensitive) a different pattern emerges. As clearly evident in Figure 1b the CT patients whose tumours were sensitive *in vitro* lived significantly longer than those with resistant tumours (log-rank $P = 0.023$, rank-sum $P = 0.006$). The median survival times were 185 weeks for the patients with *in vitro* sensitive tumours and 31 weeks for the patients with *in vitro* resistant tumours. Of importance is that there is a statistically significant positive correlation ($r = 0.7$) between level of adriamycin induced inhibition of uridine uptake and patient survival time. This means that the more sensitive the tumour was to adriamycin the more responsive was the patient to chemotherapy treatment. At this point it is important to

Correspondence: M. Volm
Received 30 September 1987; and in revised form 12 November 1987

**Figure 1** Survival patterns of (a) all patients ($n=32$) with stage III lung adenocarcinomas subdivided according to therapy. Fourteen patients were treated with surgery alone (group S) and 18 were treated with surgery plus chemotherapy (group CT). (b) patients with stage III lung adenocarcinomas treated with surgery plus chemotherapy (group CT) and subdivided according to results using the *in vitro* short term test (sensitive, resistant). (c) patients with stage III lung adenocarcinomas treated by surgery alone (group S) and subdivided according to results of the *in vitro* short term test (sensitive and resistant).

realize that while the numbers of patients are small, the survival times of patients who were treated with surgery alone and divided according to the test results were not different (log-rank $P=0.14$, rank-sum, $P=0.24$, Figure 1c). Thus the observed differences in survival times of patients treated with surgery plus chemotherapy according to the test results (sensitive–resistant) may be largely attributed to the drug therapy.

The age, pT, pN, tumour size and surgery procedures were similar in all groups (Table I). The distribution of treatment procedures (A, B, C) is similar in both groups of patients treated with chemotherapy. Thus, it appears that the *in vitro* short term test was successful *a priori* in predicting which patients might respond significantly to a chemotherapy regimen. While we used only adriamycin to predict the tumour response, the treatment protocols varied considerably and therefore, further studies are required to show whether multidrug-resistance or other reasons are responsible for this phenomenon. Should these results be supported by other clinical studies, it is quite possible that more effective therapies might be designed for patients with *in vitro* sensitive tumours. Similarly, those patients with *in vitro* resistant tumours might be better served by entirely new strategies, i.e. hyperthermia or immunotherapy.

**Table I** Clinical characteristics within the groups of patients

| Group | Number pts | Age | pT 2 | pT 3 | pN 0 | pN 1 | pN 2 | Tumour size[a] ($cm^3$) | Surgery L | Surgery P | Surgery R | CT A | CT B | CT C |
|---|---|---|---|---|---|---|---|---|---|---|---|---|---|---|
| S | 14 | 61± 5 | 1 | 13 | 6 | 3 | 5 | 475± 947 | 5 | 5 | 4 | – | – | – |
| CT | 18 | 56±10 | 3 | 15 | 2 | 3 | 13 | 589± 997 | 10 | 3 | 5 | 5 | 8 | 5 |
| $CT_{sens}$ | 8 | 54± 7 | 1 | 7 | 0 | 0 | 8 | 520± 911 | 4 | 2 | 2 | 2 | 4 | 2 |
| $CT_{res}$ | 10 | 56±13 | 2 | 8 | 2 | 3 | 5 | 668±1132 | 6 | 1 | 3 | 3 | 4 | 3 |
| $S_{sens}$ | 5 | 62± 6 | 0 | 5 | 1 | 2 | 2 | 590±1096 | 3 | 2 | – | – | – | – |
| $S_{res}$ | 9 | 60± 5 | 1 | 8 | 5 | 1 | 3 | 394± 929 | 2 | 3 | 5 | – | – | – |

S = surgery alone, CT = surgery plus chemotherapy, sens = sensitive, res = resistant, L = lobectomy, P = pneumonectomy, R = resection, A, B, C = chemotherapy protocols (see text); [a]mean ± s.d.

### References

BECH-HANSEN, N.T., TILL, J.E. & LING, V. (1976). Pleiotropic phenotype of colchicine-resistant CHO cells: Cross-resistance and collateral sensitivity. *J. Cell. Physiol.*, **88**, 23.

COX, D.R. (1972). Regression models and life tables. *J. Roy. Statist. Soc B* **34**, 187.

EDLER, L., WAHRENDORF J. & BERGER, J. (1980). Survival – a program package for the statistical analysis of censored survival times. *Statist. Software Newslett.*, **6**, 44.

GEHAN, E.A. (1965). A generalized Wilcoxon test for comparing arbitrarily singly censored samples. *Biometrika*, **52**, 203.

GROUP FOR SENSITIVITY TESTING OF TUMOURS (KSST) (1981). *In vitro* short-term test to determine the resistance of human tumors in chemotherapy. *Cancer*, **48**, 2127.

KAPLAN, E.L. & MEIER, P. (1958). Nonparametric estimation from incomplete observations. *J. Am. Statist. Assoc.*, **53**, 457.

LEGHA, S.S., MUGGIA, F.M. & CARTER, S.K. (1977) Adjuvant chemotherapy in lung cancer. *Cancer*, **9**, 1415.

MATTERN, J. & VOLM, M. (1982). Clinical relevance of predictive tests for cancer chemotherapy. *Cancer Treat. Rev.*, **9**, 267.

SALMON, S.E., HAMBURGER, A.W., SOEHNLEIN, B., DURIE, B.G.M., ALBERTS, D.S. & MOON, T.E. (1978). Quantitation of differential sensitivity of human-tumor stem cells to anticancer agents. *N. Engl. J. Med.*, **298**, 1321.

STRAUS, M.J., SELAWRY, O.S. & WALLACH, R.A. (1983). Chemotherapy in lung cancer. In *Lung Cancer, Clinical Diagnosis and Treatment*, Straus, M.J. (ed) p. 261. Grune and Stratton, New York.

VOLM, M., WAYSS, K., KAUFMANN, M. & MATTERN, J. (1979). Pretherapeutic detection of tumour resistance and the results of tumour chemotherapy. *Eur. J. Cancer*, **15**, 983.

VOLM, M. (1984). Use of tritiated nucleotide incorporation for prediction of sensitivity of tumors to cytostatic agents. *Behring Inst. Mitt.*, **74**, 273.

WORLD HEALTH ORGANIZATION (1981). Histological typing of lung tumours. *Tumori*, **6**, 253.